# Spectral Precision: The Added Value of Dual-Energy CT for Axillary Lymph Node Characterization in Breast Cancer

**DOI:** 10.3390/cancers18030363

**Published:** 2026-01-23

**Authors:** Susanna Guerrini, Giulio Bagnacci, Paola Morrone, Cecilia Zampieri, Chiara Esposito, Iacopo Capitoni, Nunzia Di Meglio, Armando Perrella, Francesco Gentili, Alessandro Neri, Donato Casella, Maria Antonietta Mazzei

**Affiliations:** 1 Diagnostic Imaging Unit, Department of Medical Sciences, Azienda Ospedaliero Universitaria Senese, 53100 Siena, Italy; 2 Department of Medical, Surgical and Neuro Sciences, University of Siena, 53100 Siena, Italy; 3 Breast Cancer Surgery Unit, Department of Women’s and Children’s Health, University of Siena, Azienda Ospedaliero-Universitaria Senese, 53100 Siena, Italy

**Keywords:** dual-energy CT, breast cancer, medical imaging, precision medicine, prediction model, lymph node

## Abstract

**Background/Objectives**: To develop and validate a predictive model that combines morphological features and dual-energy CT (DECT) parameters to non-invasively distinguish metastatic from benign axillary lymph nodes in patients with breast cancer (BC). **Methods**: In this retrospective study, 117 patients (median age, 65 years; 111 women and 6 men) who underwent DECT followed by axillary lymphadenectomy between April 2015 and July 2023, were analyzed. A total of 375 lymph nodes (180 metastatic, 195 benign) were evaluated. Two radiologists recorded morphological criteria (adipose hilum status, cortical appearance, extranodal extension, and short-axis diameter) and placed regions of interest to measure dual-energy parameters: attenuation at 40 and 70 keV, iodine concentration, water concentration and spectral slope. Normalized iodine concentration was calculated using the aorta as reference. Univariate analysis identified variables associated with metastasis. Multivariate logistic regression with cross-validation was used to construct two models: one based solely on morphological features and one integrating water concentration. **Results**: On univariate testing, all DECT parameters and morphological criteria differed significantly between metastatic and benign nodes (*p* < 0.01). In multivariate analysis, water concentration emerged as the only independent DECT predictor (odds ratio = 0.97; *p* = 0.002) alongside cortical abnormality, absence of adipose hilum, extranodal extension and short-axis diameter. The morphologic model achieved an area under the receiver operating characteristic curve (AUC) of 0.871. Increasing water concentration increased the AUC to 0.883 (ΔAUC = 0.012; *p* = 0.63, not significant), with internal cross-validation confirming stable performance. **Conclusions**: A model combining standard morphologic criteria with water concentration quantification on DECT accurately differentiates metastatic from benign axillary nodes in BC patients. Although iodine-based metrics remain valuable indicators of perfusion, water concentration offers additional tissue composition information. Future multicenter prospective studies with standardized imaging protocols are warranted to refine parameter thresholds and validate this approach for routine clinical use.

## Simple Summary

Accurate assessment of lymph node involvement is crucial for tailoring breast cancer treatment. This study explores an advanced imaging approach that combines dual-energy CT (DECT) with morphological features to distinguish benign from cancer-affected lymph nodes. By analyzing both the physical appearance of nodes and their chemical composition after contrast injection, our model can support non-invasive identification of metastatic nodes. Although iodine concentration remains informative, our findings show that water concentration provides complementary diagnostic information. Morphologic features, however, remain the cornerstone for nodal assessment. This integrated imaging strategy can enhance surgical planning, reduce unnecessary procedures and improve patient outcomes. Future large-scale studies are needed to standardize protocols and confirm these findings across diverse patient populations.

## 1. Introduction

Breast cancer (BC) represents the most commonly diagnosed malignancy in women globally and remains the foremost cause of cancer-related mortality (11.6% incidence, 6.9% mortality) [1]. Accurate staging of the axillary lymph nodes (LNs) status is critical to guide treatment planning and improve the prospects for cure and survival [2,3]; identifying axillary metastatic involvement is essential for guiding decisions regarding systemic chemotherapy or radiotherapy.

Clinically, the two principal prognostic determinants in breast cancer are the presence of axillary lymph node metastases and the size of the primary tumor [4]. Axillary nodal involvement is well recognized as a marker of unfavorable prognosis, with a reported reduction in 5-year survival of approximately 28% to 40% in affected patients [5].

Axillary lymph node dissection (ALND) represents the definitive technique for confirming axillary metastasis; however, sentinel lymph node biopsy (SLNB) has largely replaced it as the standard approach for axillary assessment in early-stage breast cancer. This shift reflects the substantially lower morbidity associated with SLNB compared with ALND [6,7].

Until recently, patients with positive SLNB were subjected to ALND, but today this practice is being questioned [8,9,10].

However, this reduction in axillary surgery is not universally accepted, as omitting ALND may result in a lack of information on the number and extent of axillary metastases, which is essential for choosing post-surgical therapies, particularly in younger patients and in the neoadjuvant setting [11].

An accurate method of preoperative identification of metastatic lymph nodes is therefore of primary importance in the surgical planning of these patients.

For these reasons European Guidelines recommend imaging of the axillary region at the time of diagnosis to appropriately plan the treatment basing on the imaging findings [12].

Ultrasound (US) is the primary non-surgical method for evaluating axillary lymph nodes, although its sensitivity is somewhat constrained [13]. In breast Magnetic Resonance Imaging (MRI), the visualization of the axilla can be limited in the field of view [2,14,15,16], so lymph node assessment can be performed by CT scan, which has many well-known advantages, such as being a non-invasive method that allows for rapid whole-body staging; not the least CT can try to overcomes the limitations of SLN biopsy, which, despite its accuracy, remains invasive and carries a 5–10% false-negative rate [17] and potential complications such as lymphedema, sensory disturbances, axillary web syndrome, and reduced range of motion of the upper limb [7,17,18].

Dual-Energy Computed Tomography (DECT), which exploits the differential absorption characteristics of X-ray beams at different energies, allows morphological, quantitative, and functional analysis of tissues [19]. Specifically, DECT involves acquiring CT attenuation data at two distinct photon energy levels, enabling differentiation of materials based on their energy-dependent attenuation behavior [19]. From these data, monochromatic image sets can be generated and tissue composition can be assessed using material-decomposition algorithms that quantify the relative contribution of each constituent material [20,21]. In addition, the heightened attenuation of iodine at lower energies—near its k-edge of 33.2 keV—enhances visualization of vascularized lesions and improves contrast between hypervascular or hypovascular lesions and the normally enhancing parenchyma of various organs; since interstitial water content reflects microstructural and vascular alterations associated with metastasis, water content maps may outperform iodine-based perfusion metrics. These features may confer a diagnostic advantage in oncologic imaging [19,22,23,24,25,26,27,28,29] and this benefit appears particularly relevant in the context of BC [12,19,30,31,32,33]. Furthermore, DECT allows for significantly less contrast agent to be used compared to conventional CT, while maintaining the same diagnostic accuracy [34,35,36,37,38].

The purpose of this study is to identify a predictive model that combines morphological parameters and parameters derived from Dual-Energy tissue analysis, in order to characterize and predict lymph node status during staging and therefore differentiate metastatic from benign lymph nodes. This approach could improve preoperative staging and reduce the need for invasive procedures.

## 2. Materials and Methods

This retrospective study received approval from our institutional review board, which waived the need for patient authorization or informed consent owing to the use of anonymized imaging data.

### 2.1. Patients

**Figure 1:**
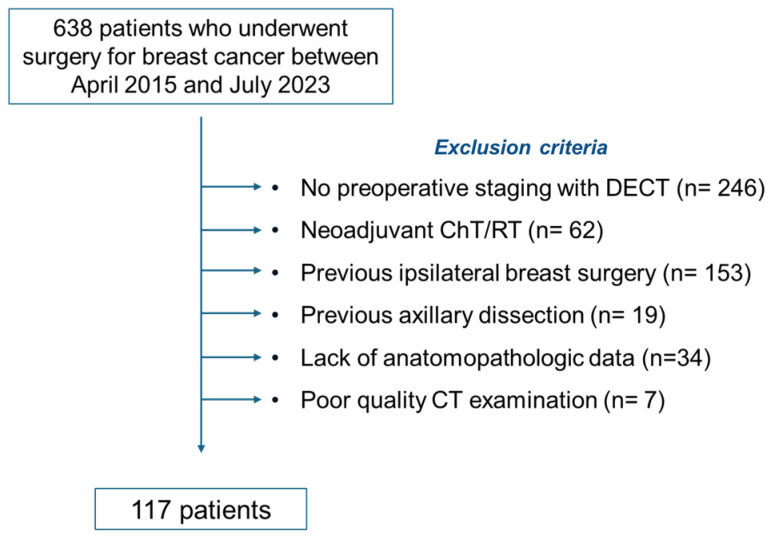
Workflow of patient selection.

A total of 638 consecutive patients who underwent surgery for BC at our center between April 2015 and July 2023 were retrospectively screened. Of these, patients without preoperative DECT of the chest, with previous axillary surgery, without histopathological confirmation of axillary lymph node status, with non-diagnostic image quality, or with incomplete clinical/pathological data were excluded from the analysis. The detailed patient selection workflow, including the number of patients excluded for each reason, is shown in Figure 1.

### 2.2. CT Imaging Acquisition

All CT examinations were obtained with the patient in the supine position, using a 64-MDCT scanner (Discovery CT 750 HD, GE Healthcare, Chicago, IL, USA). Each patient received 1.5 mL of the nonionic contrast material iopamidol (370 mg I/mL; Iopamiro 370, Bracco, Milan, Italy) per kilogram of body weight via intravenous injection through an 18-gauge needle placed in the antecubital vein, administered by a semiautomated power injector at a rate of 3–3.5 mL/s.

Each patient was instructed to hold their breath during the CT examination to prevent motion artifacts. Chest scanning, from the base of the lungs to the supraclavicular region, was performed during the late arterial phase (45–50 s after contrast medium injection) using DECT gemstone spectral imaging mode, with the following technical parameters: helical mode; rapid tube voltage switching between 80 and 140 kVp; revolution time, 0.6 s; tube current, 640 mA; beam pitch, 0.984:1; slice thickness, 2.5 mm; reconstruction interval, 1 mm; and collimation, 40 mm.

Subsequently, abdominal and brain scans were obtained using conventional CT mode parameters: helical mode for the abdomen and axial mode for the brain; tube voltage, 120 kVp; revolution time, 0.5 s; 250–500 Quality Reference mAs (GE Healthcare); slice thickness, 2.5 mm; reconstruction interval, 1 mm; and collimation, 40 mm.

Virtual monochromatic images at energies ranging from 40 to 140 keV, along with iodine maps (IC), water content maps (WC), and effective atomic number (Zeff) images, were reconstructed.

### 2.3. Image Analysis

All CT examinations of the thorax and breast were independently analyzed using AW VolumeShare7™ software v4.7 (GE HealthCare), by two radiologists, each with 3 years of experience in oncologic imaging.

### 2.4. Morphological Analysis

For morphological analysis, the long axis of the neoplasm, as well as the long axis, short axis, location and morphological features of the lymph nodes, were evaluated. These features included the presence or absence of an adipose hilum (LN adipose hilum), the normality or abnormality of the cortical area (LN cortex), and the presence or absence of extranodal extension (LN ENE).

The nodal hilum was considered absent when the central low-density fatty tissue was no longer visible. A nodal cortex was classified as abnormal when there was no high-density C-shaped rim and the cortex appeared concentric, eccentric or irregular. Extranodal extension was defined by the presence of increased density and perinodal stranding of the axillary fat [27,37,38,39,40].

All parameters were assessed independently and blindly by the two radiologists. Disagreements were resolved by a third radiologist with 10 years of experience in oncologic imaging.

### 2.5. Quantitative Analysis

For quantitative analysis, oval or circular regions of interest (ROIs) were placed on the neoplasm and axillary lymph nodes, encompassing the largest and most homogeneous areas of post-contrast enhancement. Particular care was taken to include the entire sentinel lymph node (SLN), excluding the fatty tissue of the nodal hilum and adjacent structures. To achieve this, a threshold was applied to suppress negative Hounsfield Unit (HU) values.

For each ROI, the following parameters were recorded: mean attenuation at 40 keV and 70 keV, iodine concentration (IC), water concentration (WC) and effective atomic number (Zeff).

A reference ROI was also placed on the ascending aorta [40]. IC and Zeff values of each lymph node (IC_LN and Zeff_LN) were normalized to those of the aorta (IC_aorta and Zeff_aorta), using the following formulas:

Normalized IC (NIC) = IC_LN/IC_aorta; Normalized Zeff = Zeff_LN/Zeff_aorta.

The spectral HU curve was derived from monochromatic images acquired between 40 and 140 keV. Its slope (λHU) was calculated as: λHU (HU/keV) = (HU_40 keV − HU_70 keV)/30 where HU_40 keV and HU_70 keV represent attenuation values at 40 and 70 keV, respectively [40].

Finally, the rate of difference (ROD) was calculated as described by Terada et al., using the formula:

ROD = (Lymph Node Value − Primary Lesion Value)/Primary Lesion Value.

This calculation was applied to all quantitative parameters (HU at 40 and 70 keV, IC, WC, and λHU).

A reproducibility assessment was performed on a sub cohort of 50 lymph nodes.

### 2.6. Surgery and Pathological Analysis

To ensure accurate correspondence between lymph nodes evaluated on imaging and those examined histopathologically, the operating surgeon was directly involved in the imaging review process. Intraoperative localization of sentinel nodes was achieved by the use of blue dyes or radioactive tracer and indocyanine green dyes.

During surgery, sentinel lymph nodes were identified and all resected nodes underwent intraoperative frozen section analysis, followed by standard paraffin-embedded histopathological examination.

If frozen section results were negative but paraffin analysis revealed metastatic involvement, axillary surgery or radiotherapy was performed based on the extent of disease (isolated tumor cells, micrometastases, or macrometastases). All patients subsequently received standardized adjuvant therapy according to multidisciplinary team recommendations.

Surgical approaches included either mastectomy or breast-conserving surgery, based on tumor characteristics and patient-specific factors. All decisions were taken after multidisciplinary discussion.

Histopathological data were extracted from final surgical pathology reports and included: tumor size, pathological T and N stage, histologic subtype, tumor grade, estrogen receptor (ER) status, progesterone receptor (PR) status, HER2 expression, and Ki-67 proliferation index. Lymph node staging was based on the AJCC Cancer Staging Manual (8th edition): lymph nodes containing isolated tumor cells, defined as clusters ≤ 0.2 mm or ≤200 tumor cells, were considered node-negative, whereas those with micrometastases (≤2 mm) or macrometastases were considered node-positive (pN1 or higher).

### 2.7. Statistical Analysis

Descriptive statistics were reported as medians and interquartile ranges (IQR) for continuous variables and as absolute frequencies and percentages for categorical variables. For univariate comparisons continuous variables were assessed using the Mann–Whitney U test or T-student test after assessing distributions with Shapiro–Wilk test, while categorical variables were compared using chi-square or Fisher’s exact test, as appropriate. Prior to multivariable modeling, variance inflation factors (VIFs) were calculated to assess multicollinearity; variables with VIF > 5 were excluded to ensure model stability. Logistic regression models were developed to predict nodal status. To include variables into the model a *p* value < 0.01 was considered to reduce the number of variables and facilitate interpretability. Model coefficients were reported as odds ratios (ORs) with 95% confidence intervals and associated *p*-values. Discriminative performance was evaluated using the area under the receiver operating characteristic curve (AUC), with internal validation based on repeated stratified 5-fold cross-validation. The DeLong test was applied to compare AUCs between models. Final ROC curves were plotted for each model and for individual predictors to facilitate visual comparison. A two-sided *p*-value < 0.01 was considered statistically significant.

Reproducibility analysis included ICC for quantitative variables and kohen’s kappa for qualitative ones.

Statistical analyses were performed using R (v4.4.1) and SPSS (v26).

## 3. Results

Following the application of exclusion criteria, 117 patients (111 females and 6 males), with a median age of 65 years (IQR: 53–76), were included in the study. Based on pathological results, 67 patients were classified as node-positive (N+) and 50 as node-negative (N−). Demographic and anatomo-pathological characteristics are detailed in Table 1.

Patients were grouped according to nodal status (N0 vs. N+). Within the N+ group, 36 (30.8%) were N1, 20 (17.1%) N2 and 11 (9.4%) N3. A higher T stage, larger tumor size and older age were significantly associated with the presence of pathological lymph nodes.

For each patient, at least one axillary lymph node was analyzed, yielding a total of 375 nodes (mean 3.2 ± 0.6 per patient): 180 were pathological and 195 benign. Of these, 366 nodes were located at axillary level I, 9 at level II. Median ROI dimension was 15,85 mm^2^ (IQR 9.6–32.3 mm^2^).

At univariate analysis, significant differences (*p* < 0.01) were observed between pathological and benign lymph nodes in attenuation values at 40 keV and 70 keV, spectral HU slope, IC, WC and long and short axis dimensions (Table 2). Reproducibility analysis showed good to excellent agreement between readers for long axis, short axis and node DECT derived parameters (ICC 0.82–0.95) while the RODs between primary lesion and lymph nodes showed poor to moderate agreement (ICC 0.43–0.65).

Morphological parameters including cortical status, ENE and presence of adipose hilum also showed significant associations (*p* < 0.01) with lymph node status (Table 3). Reproducibility analysis showed good agreement (Cohen’s *κ* > 0.80).

A logistic regression model was constructed including all variables with *p* < 0.01. The final model selected short axis, average WC, cortical status, adipose hilum and ENE (Table 4).

**Figure 2:**
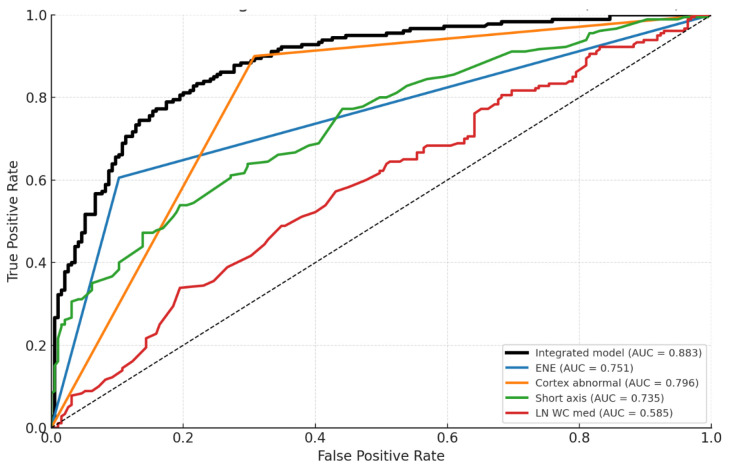
Integrated model vs. Single predictors.

The corresponding ROC curve showed an AUC of 0.883, indicating good discriminatory power (Figure 2).

Model performance was internally validated using 5 × 10-fold repeated cross-validation (mean AUC: 0.883 ± 0.042).

**Figure 3:**
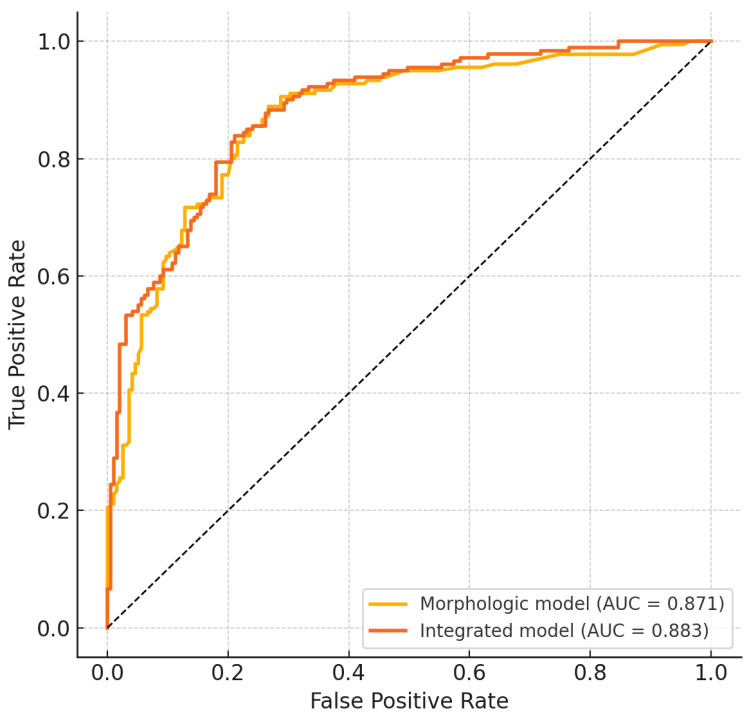
Comparison of Non-DECT and Integrated models.

An alternative model excluding contrast-enhanced CT parameters was also developed, yielding an AUC of 0.871 (Table 5 and Figure 3).

The inclusion of contrast in the model yielded a gain in diagnostic accuracy (ΔAUC = 0.012); however, no statistically significant difference was observed between the two models (*p* = 0.629).

## 4. Discussion

Our analysis demonstrates that, amongst multiple DECT parameters and morphological features, cortical irregularity, absence of adipose hilum, ENE, short-axis diameter and post-contrast WC, independently predict nodal metastasis. Univariate testing confirmed significant associations for NIC and spectral slope, but multivariate modeling identified WC alongside morphologic criteria as the strongest discriminators. Both logistic models performed similarly (ΔAUC = 0.012), indicating that morphologic criteria already capture most discriminative information, while WC provides only modest incremental value.

Recent advances in DECT have underscored its potential to non-invasively distinguish between benign and metastatic axillary lymph nodes in BC patients [41,42,43]. Compared to prior work, our integrated DECT model yields robust, but not superior, results. The study by Zhang et al. [27] reported an accuracy of 90.5% for venous-phase spectral slope in sentinel lymph nodes, exceeding our combined model’s performance and indicating that broader nodal cohorts may benefit variably from perfusion metrics. Similarly, Terada et al. [29] demonstrated high rates of difference (ROD) between primary tumors and nodes; in our cohort, ROD measures did not achieve comparable significance, underscoring the need to temper expectations about perfusion-based DECT biomarkers across diverse patient populations.

Kong et al. have posited that DECT offers substantial promise in differentiating metastatic from benign lymph nodes by leveraging NIC and spectral slope measurements in the arterial phase but advocate for rigorous prospective cohort studies with standardized protocols and the integration of morphological features to refine parameter combinations [44]. Our comprehensive evaluation interrogates these recommendations by systematically comparing contrast-enhanced DECT parameters and non-contrast parameters alongside established morphologic criteria.

The analysis revealed significant differences between metastatic and non-metastatic LNs for several DECT-derived parameters, including attenuation values at 40 and 70 keV, IC and WC, and Zeff, corroborating findings from previous research [23,24,25,26]. Particularly, the IC and slope of the spectral HU curve (between 40 and 70 keV) emerged as reliable markers of malignancy, reflecting the increased vascularity and cellularity typically associated with metastatic nodes (Table 3).

Conversely, when subjected to multivariate logistic regression (Table 4), WC emerged as the sole DECT-derived parameter retaining independent significance (OR = 0.972; *p* < 0.01), superseding IC. Post-contrast WC likely reflects interstitial edema, a key pathologic feature of reactive nodes, by quantifying water-equivalent attenuation. Experimental synthetic MRI and optoacoustic studies [45,46] have shown elevated water signals in benign nodes due to stromal edema and sinus histiocytosis; however, these techniques remain largely exploratory and not directly transferable to clinical DECT workflows. By integrating WC mapping, DECT leverages existing contrast-enhanced acquisitions to extract complementary information about nodal microenvironment without relying on specialized modalities.

To elucidate the relative contributions of contrast-dependent and morphological indicators, we constructed two predictive models. The non-contrast-enhanced (morphologic) model (Table 5) incorporated adipose hilum preservation, cortical architecture, ENE and short-axis diameter, achieving an AUC of 0.871. The integrated model augmented these morphologic variables with WC, yielding an AUC of 0.883 (Figure 3).

The Node Reporting and Data System (Node-RADS) is the standardized morphological scoring system for lymph node assessment and is currently the most widely used criteria in the literature for lymph node characterization. However, Node-RADS does not consider contrast medium among its diagnostic criteria, focusing instead on size, shape, hilum, and extra-nodal features [47]. In our study, the morphologic model was based on four CT-derived variables: short-axis diameter, cortical morphology, fatty hilum status and extranodal extension. These parameters directly correspond to the core Node-RADS descriptors “size”, “cortex/shape”, “hilum” and “extranodal features”, although some Node-RADS items were not systematically collected in our retrospective cohort. Our results indicate that the addition of post-contrast WC produces marginal but significant improvements (ΔAUC = 0.012) over Node-RADS morphology alone; integrating DECT parameters such as WC into Node-RADS may enable a semi-quantitative extension of this system, potentially standardizing spectral lymph node evaluation.

**Figure 4:**
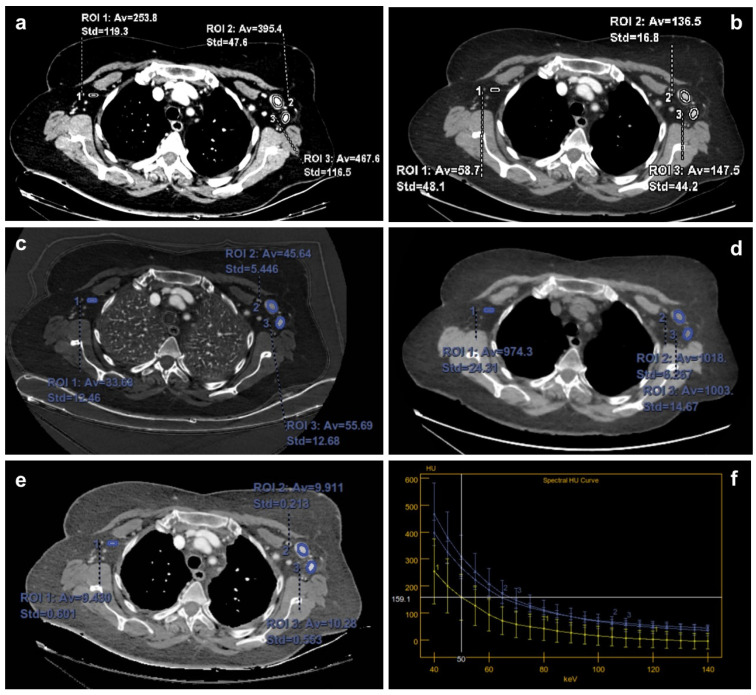
Representative DECT images in Patient with left BC. Oval or circular regions of interest (ROIs) were placed on the benign (ROI 1) and metastatic (ROI 2 and 3) axillary lymph nodes encompassing an area of contrast enhancement on DECT scan, as large and homogeneous as possible (**a**); the ROI values represent the average density value (HU). Then for each ROI we recorded the mean attenuation value at 40 keV (**a**) and 70 keV (**b**), iodine concentration (IC, (**c**)), water concentration (WC, (**d**)) and effective Z value (Zeff, (**e**)). DECT images showed that WC of the spectral Hounsfield unit curve (WHu) were significantly different in benign (ROI 1) and metastatic lymph nodes (ROI 2 and ROI 3), (**f**). The WHu of the metastatic lymph node is higher than that of the benign lymph node.

From a pathophysiological point of view, metastatic infiltration compromises nodal trabecular integrity and erases the adipose hilum, causing a reduction in interstitial compliance and an increase in water retention capacity, which leads to an increase in WC values on DECT. In contrast, reactive nodes show stromal edema and sinusoidal histiocytosis, with non-significant WC values at DECT. By quantifying the water-equivalent attenuation coefficient, DECT material decomposition maps provide a surrogate marker for these microenvironmental changes, offering an objective complement to morphological assessment. Morphological descriptors (hilum status, cortical thickness, and ENE) remain fundamental for node assessment due to their high specificity. Our results reinforce the idea that the integration of WC improves the robustness of the model (Figure 4), [34,48,,49].

However, certain limitations must be acknowledged. The retrospective nature of the study and the single-center design may introduce selection bias. Although interobserver agreement was high (Cohen’s κ > 0.8), manual ROI placement remains a source of variability. The analysis was performed at the node level and assumed independence of observations, while multiple nodes per patient may have led to a modest underestimation of variability and slightly overoptimistic performance estimates. The acquisition of a single late arterial phase may result in a possible loss of information from the IC, as well as from other DECT parameters related to perfusion. Finally, since the vast majority of evaluated lymph nodes were located at level I, our results mainly reflect the performance of DECT for level I axillary nodes and cannot be readily extrapolated to different lymph nodes. Future multicenter prospective studies with automated segmentation methods could enhance reproducibility and generalizability.

In alignment with Kong et al.’s recommendations, future investigations should focus on multicenter trials employing harmonized DECT acquisition parameters, automated or semi-automated segmentation for reproducible ROI delineation and the exploration of tumor-specific threshold values for both WC and iodine metrics. Such efforts will be critical to establishing DECT-based protocols that deliver precision-guided lymph node assessment across oncologic subtypes. Integrating WC into standardized morphological frameworks like Node-RADS may represent the next step toward reproducible, quantitative nodal characterization.

## 5. Conclusions

This study demonstrates that integrating DECT-derived WC with morphological features improves differentiation between benign and metastatic lymph nodes in BC. Morphological characteristics remain the basis for the assessment of pathological lymph nodes. Although IC and spectral slope remain informative about lymph node perfusion, the addition of WC produced only a modest improvement in diagnostic accuracy when included in the integrated diagnostic model. Moving forward, multicenter trials with harmonized DECT protocols and automated segmentation are needed to refine parameter thresholds and optimize combined morphologic and spectral models; incorporating WC into preoperative staging protocols may reduce reliance on sentinel node biopsy in selected patients.

## Figures and Tables

**Table 1:** Demographic, pathological and biomolecular variables.

Variable	TOT	N+	N0	*p*-Value
**Age (years)**	65 (53–76)	70 (56–79)	59 (52–69)	**0.005**
**T Diameter (mm)**	22 (15–35)	27 (19–43)	16 (13–23)	**<0.001**
**Surgery**				
Mastectomy	79 (67.5%)	49 (73.1%)	30 (60.0%)	0.164
Quadrantectomy	38 (32.5%)	18 (26.9%)	20 (40.0%)	
**Grading**				
G1	16 (13.7%)	4 (6.0%)	12 (24.0%)	0.354
G2	68 (58.1%)	46 (68.7%)	22 (44.0%)	
G3	32 (27.4%)	17 (25.4%)	15 (30.0%)	
**Histotype**				
Mixed	3 (2.6%)	1 (1.5%)	2 (4.0%)	0.172
Ductal	71 (60.7%)	45 (67.2%)	26 (52.0%)	
Infiltrant	15 (12.8%)	9 (13.4%)	6 (12.0%)	
Lobular	28 (23.9%)	12 (17.9%)	16 (32.0%)	
**cT**				
T1	57 (48,7%)	21 (31,3%)	36 (72.0%)	**<0.001**
T2	43 (36.8%)	31 (46.3%)	12 (24.0%)	
T3	10 (8.5%)	8 (11.9%)	2 (4.0%)	
T4	7 (6.0%)	7 (10.4%)	0 (0.0%)	
**Ki67**				
<15%	27 (23.1%)	12 (17.9%)	15 (30.0%)	0.25
15–30%	63 (53.8%)	40 (59.7%)	23 (46.0%)	
>30%	27 (23.1%)	15 (22.4%)	12 (24.0%)	
**Her-2**				
neg	92 (78.6%)	54 (80.6%)	38 (76.0%)	0.709
1+	6 (5.1%)	2 (3.0%)	4 (8.0%)	
2+	13 (11.1%)	6 (9.0%)	7 (14.0%)	
3+	6 (5.1%)	5 (7.5%)	1 (2.0%)	
**PgR**				
<10%	34 (29.1%)	16 (32.0%)	18 (26.9%)	0.681
≥10%	83 (70.9%)	34 (68.0%)	49 (73.1%)	
**ER**				
<1%	20 (17.1%)	10 (14.9%)	10 (20.0%)	0.636
≥10%	97 (82.9%)	57 (85.1%)	40 (80.0%)	

**Table 2:** Univariate analysis of quantitative parameters.

Variable	LN_NEG (MEAN ± SD)	LN_POS (MEAN ± SD)	*p*-Value
**Long axis (mm)**	10.00 ± 5.29	12.17 ± 6.74	**<0.001**
**Short axis (mm)**	4.93 ± 1.85	7.85 ± 4.54	**<0.001**
**40 kev med (HU)**	275.02 ± 67.55	304.04 ± 86.01	**<0.001**
**40 kev std.dev (HU)**	45.92 ± 19.20	49.72 ± 19.24	0.057
**70 kev med (HU)**	81.04 ± 23.64	93.32 ± 28.98	**<0.001**
**70 kev std.dev (HU)**	16.87 ± 6.59	17.93 ± 6.67	0.123
**HU Slope**	6.47 ± 1.65	7.02 ± 2.09	**0.005**
**IC med**	33.32 ± 8.45	36.15 ± 10.27	**0.004**
**IC ds**	5.36 ± 2.23	5.90 ± 2.24	0.021
**Eff-Z med**	9.42 ± 0.39	9.53 ± 0.45	0.012
**Eff-Z std.dev**	0.24 ± 0.10	0.26 ± 0.09	0.036
**WC med**	1000.30 ± 18.04	1005.40 ± 17.31	**0.005**
**WC std.dev**	8.76 ± 2.84	8.94 ± 2.64	0.524
**NIC**	0.41 ± 0.16	0.47 ± 0.45	0.068
**NE ff-Z**	0.83 ± 0.07	0.83 ± 0.07	0.625
**ROD 40 keV**	0.52 ± 0.75	0.56 ± 0.63	0.523
**ROD 70 keV**	0.22 ± 0.72	0.24 ± 0.57	0.680
**ROD IC**	1.06 ± 2.61	0.88 ± 0.90	0.362
**ROD WC**	−0.02 ± 0.02	−0.02 ± 0.02	0.746
**ROD Slope**	0.74 ± 0.88	0.82 ± 0.86	0.403

**Table 3:** Univariate analysis of morphological parameters.

Variable	LN_NEG	LN_POS	*p*-Value
**Adipose hilum**			
No	47	129	**<0.001**
Yes	148	51	
**Cortex**			
Abnormal	60	162	**<0.001**
Normal	135	18	
**ENE**			
No	175	71	**<0.001**
Yes	20	109	

**Table 4:** Integrated model.

Variable	OR	IC 2.5%	IC 97.5%	*p*-Value
**ENE**	3.546	1.812	6.939	<0.001
**Cortex**	7.491	3.596	15.607	<0.001
**Short axis**	1.248	1.093	1.426	0.001
**WC med**	0.972	0.956	0.990	0.002

**Table 5:** Non-contrast-enhanced model.

Variable	OR	CI 2.5%	CI 97.5%	*p*-Value
**ENE**	3.043	1.581	5.857	<0.001
**Cortex**	5.868	2.932	11.747	<0.001
**Adipose hilum**	0.442	0.246	0.794	0.006
**Short axis**	1.169	1.038	1.316	0.010

## Data Availability

The raw data supporting the conclusions of this article will be made available by the authors on request.

## Abbreviations

The following abbreviations are used in this manuscript:

BC	Brest Cancer
LNs	Lymph Nodes
ALND	Axillary Lymph Node Dissection
SLN	Sentinel Lymph Node Biopsy
US	Ultrasound
MRI	Magnetic Resonance Imaging
CT	Computed Tomography
DECT	Dual-Energy CT
ENE	Extranodal Extension
ROI	Region of Interest
IC	Iodine Concentration
WC	Water Concentration
Zeff	Effective Z value
HU	Hounsfield Unitk
